# Detection of rabies viral neutralizing antibodies in the Puerto Rican *Brachyphylla cavernarum*

**DOI:** 10.1080/20008686.2020.1840773

**Published:** 2020-10-29

**Authors:** Andrew Hirsbrunner, Armando Rodriguez-Duran, Jodie A. Jarvis, Robert J. Rudd, April D. Davis

**Affiliations:** a Wadsworth Center Rabies Laboratory, New York State Department of Health, Slingerlands, NY, USA; b Universidad Interamericana de Puerto Rico, Bayamón, Mata de Plátano Field Station, Bayamón, Puerto Rico

**Keywords:** *Brachyphylla cavernarum*, neutralizing antibodies, Puerto Rico, rabies

## Abstract

The purpose of this study was to determine if Puerto Rican bats had previous exposure to rabies virus based on viral neutralizing antibodies. Our results demonstrate that 6.5% of the bats in this study had some exposure to rabies virus. The route of exposure is unknown but may have occurred following interaction with a rabid terrestrial animal or an unidentified bat rabies virus.

Puerto Rico is home to 13 diverse species of bats, none of which is currently considered rabies vector species, but their capacity to become a vector is unknown [1]. Despite this, the possibility exists that bats may be exposed to rabies through species introduced to Puerto Rico in which rabies is endemic, such as mongooses. Spill over from mongooses occurs in terrestrial animals including dogs and cats [2]. Additionally, rabies-infected animals from neighboring countries could be transported to the island via ship or weather event. Although the CDC traveler’s health website states bat rabies can be found in Puerto Rico, bat rabies has never been documented in the US territory (CDC.gov). However, the lack of surveillance prevents the definitive classification as free of bat rabies.

The purpose of this study was to determine the presence of anti-rabies neutralizing antibodies (rVNA) in Antillean fruit-eating bats (*Brachyphylla cavernarum*). Two hundred eighteen healthy Antillean fruit-eating bats, a common non-migratory species of bat found throughout Puerto Rico and the Lesser Antilles, were captured from Cueva Bonita Cave in north-central Puerto Rico from 2012 to 2014 (Figure 1). Bats were hand netted as described in 3 and transported to the Universidad Interamericana de Puerto Rico – Bayamón Campus laboratory. Blood collection from the propatagium vein as described in Davis et al. [4] was performed without anesthesia. Following blood collection, bats were banded as described in Kunz and Weise [5], transported back to Cueva Bonita Cave, and released. While *B. cavernarum* is not the only bat species on the island, they were chosen for several reasons; their size makes it easier to obtain samples, they form large colonies which makes it easier to obtain adequate sample sizes, and they are pugnacious and gregarious animals that live in close contact with other members of the colony.

**Figure 1.**
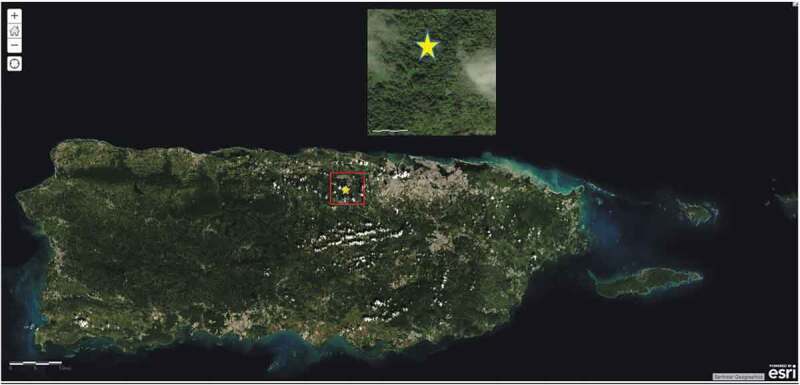
Map of Puerto Rico, insert with yellow star denoting location of the collection site as described in Gannon et al. [1]

Blood samples were sent to the Wadsworth Center Rabies Laboratory for processing. Determination of the rVNA titer was performed using the procedure described in Trimarchi et al. [6] with modifications made to account for the low sample volume. The assay compares the dilution at which the sample completely neutralizes the rabies Challenge Virus Standard (CVS-11) to the dilution which the US Standard Rabies Immune Globulin (Food and Drug Administration, Silver Spring, Maryland) achieves complete virus neutralization. Samples collected from August 2012 to February 2013 were processed using a modified serum neutralization assay, in which the volumes of all reagents used were reduced to half to decrease the required sample volume from 50 µl to 25 µl [4]. Starting serum volumes for these 117 samples ranged from 1 µl to 19 µl and growth media (Eagle’s minimum essential medium supplemented with 10% fetal bovine serum, 2 mM glutamine, 2.2 mg/ml sodium bicarbonate, 200IU/ml penicillin, and 0.4 mg/ml streptomycin; GM) were used to bring the sample to 25 µl. The volume of samples collected from December 2013 to March 2014 was doubled by adding GM to accommodate for the routine 50 µl sample volume. The dilution factors of serum samples in each method were taken into consideration when calculating rabies antibody titers.

While an rVNA titer of 0.5IU/ml is considered an adequate response for humans at high risk of rabies exposure, such as veterinarians [7], a lower titer of 0.25IU/ml is considered adequate for wildlife [8]. However, it has also been shown that an antibody response is not always detected following a survivable experimental RABV inoculation [4,9]. Consequently, an adequate response is not necessary to indicate a rabies exposure, and titers lower than 0.25IU/ml would also be indicative of an exposure. Of the 216 bats that were tested, 14 (6.5%) had an rVNA titer of at least 0.125IU/ml. Three percent (4/117) of samples collected in 2012–2013 and processed with the modified serum neutralization assay had a demonstrable titer compared to 10% (10/99) of the 2013–2014 bats (Table 1). The difference in seroconversion between the two collection periods is most likely due to sample volume size. The 2012–2013 samples required a considerable amount of GM to achieve the 25 µl of required for the modified serum neutralization assay. Consequently, the sensitivity of the modified serum neutralization assay was greatly reduced: 56% (65/117) of all the samples collected from 2012 to 2013 could not result in a titer of 0.125IU/ml due to the reduced sensitivity of the test. Of those 65 samples, 27 (23%) could not result in a titer at or below 0.5IU/ml (Supplemental table 1). When compared to the titers of serum samples collected between 2013 and 2014, three of the fourteen positive samples had a titer of 0.125IU/ml, illustrating the importance of detecting lower titers.

**Table 1.** Samples with significant titers

Sex	Date caught	ID	Starting volume (µl]	GM added (µl)	Dilution Factor	Titer (IU/ml)
N/A	2012	No tag-3	11	14	2.3	0.575
M	5–24-12	BC052412-6	4	21	6.3	3.15
M	5–29-12	BC052912-2	12	13	2.1	0.2625
M	5–29-12	BC052912-7	2	23	12.5	1.5625
F	12-1-13	BC120113-18	50	50	2.0	0.5
M	12-1-13	BC120113-27	30	30	2.0	0.25
F	12-1-13	BC120113	25	25	2.0	0.125
F	2-2-14	BC020214-9	100	100	2.0	0.25
F	2-2-14	BC020214-23	40	40	2.0	0.25
F	2-2-14	BC020214	55	55	2.0	0.125
F	2-2-14	BC020214	40	40	2.0	0.125
F	3-2-14	BC030214-17	35	35	2.0	1.0
M	3-2-14	BC030214-26	40	40	2.0	0.25
F	3-9-14	BC030914-7	40	40	2.0	0.25

Rabies antibody titers (IU/ml) of *Brachyphylla cavernarum* (BC) with antibody titers ≥0.25IU/ml. Blood samples were taken between August 2012 to February 2013 and December 2013 to March 2014 at Cueva Bonita, Puerto Rico.

Although it is unclear how bats in Puerto Rico are being exposed to rabies, there are a few hypotheses. The introduction of a rabid bat from a neighboring rabies endemic country has occurred previously on other islands. Seetahal et al. [10] reported the migration of bats between the mainland and Trinidad could likely account for the introduction of bat rabies to the island. However, given that endemic bat rabies does not occur on any of the neighboring islands, this is an unlikely scenario for Puerto Rico. Alternatively, bats could accidentally be transported via international trade or flyways [11]. In 1991 a rabid bat was found inside a shipping container that arrived in the port of Honolulu, Hawaii [12]. Puerto Rico has trade agreements with several rabies endemic countries most notably Venezuela, Mexico, Colombia, Trinidad, Brazil, and Argentina, making translocation a feasible explanation [13,14]. Other potential routes of rabies exposure could be from mongooses, feral cats, and dogs. In Puerto Rico, 17 mongooses, 11 dogs, 2 cats, and 1 small ruminant tested positive for rabies in 2017 [2]. Mongooses, cats, and dogs are well known to live in forested areas in which bat-inhabited caves are located. Both mongoose and cats are known to hunt small animals and cats have been sighted in Puerto Rican caves [15].

Although heterologous hosts are less likely to transmit rabies, it is not impossible [16]. From 2000 to 2016 the Puerto Rico Department of Health Rabies Laboratory tested 407 cats, 46 (11%) of which tested positive for rabies (courtesy of Puerto Rico Department of Health). Due to their small size and flight requirements, the ability of bats to survive an attack from a predator is believed to be unlikely. However, a small wing laceration or scratch from a tooth may be survivable for a bat.

While bat rabies is not endemic, our research demonstrates that rabies exposure has occurred in bats in Puerto Rico. However, our research does not identify the type(s) of rabid animal to which bats may be exposed. This results in a conundrum for public health practices; should humans and domestic animals with direct contact with a bat be evaluated for post-exposure rabies treatment? Additional research to determine the route by which bats are exposed to rabies and the prevalence of rVNA in other bat colonies in Puerto Rico would be valuable in shaping public health policy.

## Supplementary Material

Supplemental Material
