# Supplementary material for: Detection of rabies viral neutralizing antibodies in the Puerto Rican Brachyphylla cavernarum
Source: Infect Ecol Epidemiol. 2020 Oct 29;10(1):1840773. doi: 10.1080/20008686.2020.1840773 (PMC7598998; doi:10.1080/20008686.2020.1840773)
Supplement: Supplemental Material [file ZIEE_A_1840773_SM1078.docx]

Supplemental Table 1: All Serum samples

Rabies antibody titers (IU/ml) of *Brachyphylla cavernarum* (BC). Blood samples were taken between August 2012 to February 2013 and December 2013 to March 2014 at Cueva Bonita, Puerto Rico.

| **Sex** | **Date Caught** | **ID** | **Starting Volume (ul)** | **GM added (ul)** | **Dilution Factor** | **Titer (IU/ml)** |
| --- | --- | --- | --- | --- | --- | --- |
| M | 3-28-12 | BC032812-3 | 11 | 14 | 2.3 | <0.575 |
| M | 3-28-12 | BC032812-7 | 11 | 14 | 2.3 | <0.575 |
| M |  | BC041912-10 | 1 | 24 | 25 | <6.25 |
| M |  | BC042412-1 | 14 | 11 | 1.8 | <0.45 |
| F |  | BC042412-2 | 10 | 15 | 2.5 | <0.625 |
| M |  | BC042412-4 | 6 | 19 | 4.2 | <1.05 |
| F |  | BC042412-5 | 12 | 13 | 2.1 | <0.525 |
| F |  | BC042412-8 | 9 | 16 | 2.8 | <0.7 |
| M |  | BC042412-9 | 10 | 15 | 2.5 | <0.625 |
| F |  | BC042412-10 | 8 | 17 | 3.1 | <3.1 |
| M |  | BC051612-2 | 5 | 20 | 5 | <1.25 |
| M |  | BC051612-3 | 6 | 19 | 4.2 | <1.05 |
| M |  | BC051612-4 | 11 | 14 | 2.3 | <0.575 |
| M |  | BC051612-6 | 10 | 15 | 2.5 | <0.625 |
| M |  | BC051612-8 | 5 | 20 | 5 | <1.25 |
| M |  | BC051612-9 | 12 | 13 | 2.1 | <0.525 |
| M |  | BC052412-1 | 15 | 10 | 1.7 | <0.425 |
| M |  | BC052412-3 | 7 | 18 | 3.6 | <0.9 |
| F |  | BC052412-4 | 11 | 14 | 2.3 | <0.575 |
| M |  | BC052412-6 | 4 | 21 | 6.3 | 3.15 |
| M |  | BC052412-8 | 14 | 11 | 1.8 | <0.45 |
| M |  | BC052412-9 | 8 | 17 | 3.1 | <0.775 |
| M |  | BC052412-10 | 6 | 19 | 4.2 | <1.05 |
| M |  | BC052912-1 | 10 | 15 | 2.5 | <0.625 |
| M |  | BC052912-2 | 12 | 13 | 2.1 | 0.2625 |
| M |  | BC052912-4 | 13 | 12 | 1.9 | <0.2375 |
| M |  | BC052912-7 | 2 | 23 | 12.5 | 1.5625 |
| M |  | BC052912-8 | 8 | 17 | 3.1 | <0.3875 |
| M |  | BC052912-9 | 10 | 15 | 2.5 | <0.3125 |
| F |  | BC052912-10 | 10 | 15 | 2.5 | <0.3125 |
| F |  | BC052912-14 | 5 | 20 | 5 | <0.625 |
| M |  | BC052912-15 | 7 | 18 | 3.6 | <0.45 |
| M |  | BC061912-1 | 13 | 12 | 1.9 | <0.2375 |
| M |  | BC061912-4 | 17 | 8 | 1.5 | <0.1875 |
| F |  | BC061912-5 | 10 | 15 | 2.5 | <0.3125 |
| M | 8-7-12 | BC080712-1 | 7 | 18 | 3.6 | <0.45 |
| M | 8-7-12 | BC080712-3 | 9 | 16 | 2.8 | <0.35 |
| F | 8-7-12 | BC080712-4 | 11 | 14 | 2.3 | <0.2875 |
| M | 8-7-12 | BC080712-6 | 17 | 8 | 1.5 | <0.1875 |
| M | 8-7-12 | BC080712-7 | 7 | 18 | 3.6 | <0.45 |
| M | 8-7-12 | BC080712-8 | 8 | 17 | 3.1 | <0.3875 |
| F | 8-7-12 | BC080712-9 | 5 | 20 | 5 | <0.625 |
| F | 8-7-12 | BC080712-10 | 12 | 13 | 2.1 | <0.2625 |
| M | 8-7-12 | BC080712-12 | 11 | 14 | 2.3 | <0.2875 |
| M | 8-7-12 | BC080712-13 | 15 | 10 | 1.7 | <0.2125 |
| M | 8-16-12 | BC081612-3 | 11 | 14 | 2.3 | <0.2875 |
| M | 8-16-12 | BC081612-4 | 8 | 17 | 3.1 | <0.3875 |
| M | 8-16-12 | BC081612-5 | 11 | 14 | 2.3 | <0.2875 |
| M | 8-16-12 | BC081612-6 | 14 | 11 | 1.8 | <0.1125 |
| M | 8-16-12 | BC081612-7 | 10 | 15 | 2.5 | <0.15625 |
| M | 8-16-12 | BC081612-8 | 8 | 17 | 3.1 | <0.19375 |
| M | 8-16-12 | BC081612-9 | 8 | 17 | 3.1 | <0.19375 |
| F | 8-16-12 | BC081612-10F | 12 | 13 | 2.1 | <0.13125 |
| M | 8-16-12 | BC081612-10M | 8 | 17 | 3.1 | <0.19375 |
| M | 8-16-12 | BC081612-11 | 11 | 14 | 2.3 | <0.14375 |
| M | 8-16-12 | BC081612-12 | 13 | 12 | 1.9 | <0.11875 |
| F | 8-16-12 | BC081612-13 | 10 | 15 | 2.5 | <0.15625 |
| M | 9-11-12 | BC091112-1 | 12 | 13 | 2.1 | <0.13125 |
| M | 9-11-12 | BC091112-3 | 19 | 6 | 1.3 | <0.08125 |
| M | 9-11-12 | BC091112-4 | 7 | 18 | 3.6 | <0.225 |
| M | 9-11-12 | BC091112-5 | 13 | 12 | 1.9 | <0.11875 |
| M | 9-11-12 | BC091112-6 | 8 | 17 | 3.1 | <0.19375 |
| F | 9-11-12 | BC091112-7 | 10 | 15 | 2.5 | <0.15625 |
| F | 9-11-12 | BC091112-8 | 5 | 20 | 1.3 | <0.08125 |
| F | 9-11-12 | BC091112-9 | 6 | 19 | 4.2 | <0.2625 |
| F | 9-11-12 | BC091112-10 | 7 | 18 | 3.6 | <0.225 |
| M | 9-11-12 | BC091112-11 | 12 | 13 | 2.1 | <0.13125 |
| F | 9-11-12 | BC091112-12 | 11 | 14 | 2.3 | <0.14375 |
| F | 9-11-12 | BC091112-13 | 11 | 14 | 2.3 | <0.14375 |
| M | 9-11-12 | BC091112-14 | 15 | 10 | 1.7 | <0.10625 |
| M | 9-11-12 | BC091112-16 | 6 | 19 | 4.2 | <0.2625 |
| M | 9-11-12 | BC091112-17 | 8 | 17 | 3.1 | <0.19375 |
| F | 9-11-12 | BC091112-18 | 11 | 14 | 2.3 | <0.2875 |
| M | 9-11-12 | BC091112-19 | 8 | 17 | 3.1 | <0.3875 |
| F | 9-11-12 | BC091112-20 | 15 | 10 | 1.7 | <0.2125 |
| F | 9-11-12 | BC091112-21 | 5 | 20 | 5 | <0.625 |
| M | 9-11-12 | BC091112-22 | 10 | 15 | 2.5 | <0.3125 |
| F | 9-11-12 | BC091112-23 | 13 | 12 | 1.9 | <0.2375 |
| F | 2-6-13 | BC020613-1 | 5 | 20 | 5 | <0.625 |
| F | 2-6-13 | BC020613-2 | 17 | 8 | 1.5 | <0.1875 |
| M | 2-6-13 | BC020613-3 | 15 | 10 | 1.7 | <0.2125 |
| F | 2-6-13 | BC020613-4 | 12 | 13 | 2.1 | <0.2625 |
| M | 2-6-13 | BC020613-5 | 14 | 11 | 1.8 | <0.225 |
| F | 2-6-13 | BC020613-6 | 11 | 14 | 2.3 | <0.2875 |
| F | 2-6-13 | BC020613-7 | 15 | 10 | 1.7 | <0.2125 |
| F | 2-6-13 | BC020613-8 | 12 | 13 | 2.1 | <0.2625 |
| F | 2-6-13 | BC020613-9 | 8 | 17 | 3.1 | <0.3875 |
| F | 2-6-13 | BC020613-10 | 12 | 13 | 2.1 | <0.2625 |
| M | 2-6-13 | BC020613-11 | 13 | 12 | 1.9 | <0.2375 |
| F | 2-6-13 | BC020613-12 | 18 | 7 | 1.4 | <0.175 |
| M | 2-6-13 | BC020613-13 | 8 | 17 | 3.1 | <0.3875 |
| M | 2-6-13 | BC020613-14 | 9 | 16 | 2.8 | <0.35 |
| M | 2-6-13 | BC020613-15 | 17 | 8 | 1.5 | <0.1875 |
| F | 2-6-13 | BC020613-16 | 12 | 13 | 2.1 | <0.2625 |
| M | 2-6-13 | BC020613-18 | 8 | 17 | 3.1 | <0.3875 |
| F | 2-6-13 | BC020613-19 | 13 | 12 | 1.9 | <0.2375 |
| M | 2-6-13 | BC020613-20 | 10 | 15 | 2.5 | <0.3125 |
| F | 2-6-13 | BC020613-21 | 15 | 10 | 1.7 | <0.2125 |
| F |  | BC082812-1 | 13 | 12 | 1.9 | <0.2375 |
| M |  | BC082812-2 | 8 | 17 | 3.1 | <0.3875 |
| F |  | BC082812-3 | 5 | 20 | 5 | <0.625 |
| M |  | BC082812-4 | 5 | 20 | 5 | <0.625 |
| F |  | BC082812-5 | 17 | 8 | 1.5 | <0.1875 |
| M |  | BC082812-6 | 17 | 8 | 1.5 | <0.1875 |
| M |  | BC082812-7 | 16 | 9 | 1.6 | <0.2 |
| F |  | BC082812-9 | 14 | 11 | 1.8 | <0.225 |
| M |  | BC082812-10 | 12 | 13 | 2.1 | <0.2625 |
| M |  | BC082812-11 | 14 | 11 | 1.8 | <0.225 |
| M |  | BC082812-12 | 5 | 20 | 5 | <0.625 |
| M |  | BC082812-14 | 13 | 12 | 1.9 | <0.2375 |
| M |  | BC082812-15 | 15 | 10 | 1.7 | <0.2125 |
| M |  | no tag-8 | 14 | 11 | 1.8 | <0.225 |
| N/A |  | no tag-1 | 9 | 16 | 2.8 | <0.35 |
| N/A |  | no tag-2 | 11 | 14 | 2.3 | <0.2875 |
| N/ |  | no tag-3 | 11 | 14 | 2.3 | 0.575 |
| M |  | BC182812-12 | 12 | 13 | 2.1 | <0.2625 |
| F |  | BC091118-2 | 15 | 10 | 1.7 | <0.2125 |
| F | 12-1-13 | BC 120113-1 | 35 | 35 | 2 | <0.125 |
| F | 12-1-13 | BC 120113-2 | 20 | 20 | 2 | <0. 25 |
| M | 12-1-13 | BC 120113-3 | 30 | 30 | 2 | <0.125 |
| F | 12-1-13 | BC 120113-4 | 30 | 30 | 2 | <0.25 |
| M | 12-1-13 | BC 120113-5 | 25 | 25 | 2 | <0.25 |
| F | 12-1-13 | BC 120113-6 | 25 | 25 | 2 | 0.125 |
| F | 12-1-13 | BC 120113-7 | 40 | 40 | 2 | <0.25 |
| M | 12-1-13 | BC 120113-8 | 25 | 25 | 2 | <0.125 |
| F | 12-1-13 | BC 120113-9 | 35 | 35 | 2 | <0.25 |
| F | 12-1-13 | BC 120113-10 | 30 | 30 | 2 | <0.25 |
| F | 12-1-13 | BC 120113-11 | 50 | 50 | 2 | <0.25 |
| M | 12-1-13 | BC 120113-12 | 25 | 25 | 2 | <0.125 |
| F | 12-1-13 | BC 120113-13 | 25 | 25 | 2 | <0.25 |
| F | 12-1-13 | BC 120113-14 | 0 | 0 |  |  |
| M | 12-1-13 | BC 120113-15 | 30 | 30 | 2 | <0.125 |
| F | 12-1-13 | BC 120113-16 | 0 | 0 | 2 |  |
| F | 12-1-13 | BC 120113-17 | 40 | 40 | 2 | <0.25 |
| F | 12-1-13 | BC 120113-18 | 50 | 50 | 2 | 0.5 |
| F | 12-1-13 | BC 120113-19 | 50 | 50 | 2 | <0.125 |
| M | 12-1-13 | BC 120113-20 | 45 | 45 | 2 | <0.25 |
| F | 12-1-13 | BC 120113-21 | 35 | 35 | 2 | <0.125 |
| F | 12-1-13 | BC 120113-22 | 45 | 45 | 2 | <0.25 |
| F | 12-1-13 | BC 120113-23 | 45 | 45 | 2 | <0.125 |
| F | 12-1-13 | BC 120113-24 | 40 | 40 | 2 | <0.25 |
| M | 12-1-13 | BC 120113-25 | 35 | 35 | 2 | <0.25 |
| M | 12-1-13 | BC 120113-26 | 35 | 35 | 2 | <0.125 |
| M | 12-1-13 | BC 120113-27 | 30 | 30 | 2 | 0.25 |
| M | 2-2-14 | BC0202214-1 | 40 | 40 | 2 | <0.25 |
| F | 2-2-14 | BC0202214-2 | 45 | 45 | 2 | <0.125 |
| F | 2-2-14 | BC0202214-3 | 40 | 40 | 2 | <0.25 |
| F | 2-2-14 | BC0202214-4 | 55 | 55 | 2 | <0.125 |
| M | 2-2-14 | BC0202214-5 | 80 | 80 | 2 | <0.125 |
| F | 2-2-14 | BC020214-6 | 55 | 55 | 2 | 0.125 |
| F | 2-2-14 | BC020214-7 | 50 | 50 | 2 | <0.125 |
| M | 2-2-14 | BC020214-8 | 30 | 30 | 2 | <0.125 |
| F | 2-2-14 | BC020214-9 | 100 | 100 | 2 | 0.25 |
| M | 2-2-14 | BC020214-10 | 60 | 60 | 2 | <0.25 |
| M | 2-2-14 | BC020214-11 | 35 | 35 | 2 | <0.125 |
| F | 2-2-14 | BC020214-12 | 50 | 50 | 2 | <0.25 |
| F | 2-2-14 | BC020214-13 | 40 | 40 | 2 | 0.125 |
| M | 2-2-14 | BC020214-14 | 35 | 35 | 2 | <0.125 |
| F | 2-2-14 | BC020214-15 | 75 | 75 | 2 | <0.125 |
| M | 2-2-14 | BC020214-16 | 60 | 60 | 2 | <0.25 |
| F | 2-2-14 | BC020214-17 | 30 | 30 | 2 | <0.25 |
| M | 2-2-14 | BC020214-18 | 40 | 40 | 2 | <0.25 |
| M | 2-2-14 | BC020214-19 | 65 | 65 | 2 | <0.25 |
| F | 2-2-14 | BC020214-20 | 25 | 25 | 2 | <0.125 |
| M | 2-2-14 | BC020214-21 | 70 | 70 | 2 | <0.25 |
| F | 2-2-14 | BC020214-22 | 70 | 70 | 2 | <0.25 |
| F | 2-2-14 | BC020214-23 | 40 | 40 | 2 | 0.25 |
| M | 2-2-14 | BC020214-24 | 25 | 25 | 2 | <0.25 |
| F | 2-2-14 | BC020214-25 | 40 | 40 | 2 | <0.125 |
| M | 2-2-14 | BC020214-26 | 50 | 50 | 2 | <0.125 |
| M | 2-2-14 | BC020214-27 | 26 | 26 | 2 | <0.25 |
| F | 3-2-14 | BC030214-1 | 40 | 40 | 2 | <0.125 |
| M | 3-2-14 | BC030214-2 | 65 | 65 | 2 | <0.125 |
| M | 3-2-14 | BC030214-3 | 25 | 25 | 2 | <0.125 |
| F | 3-2-14 | BC030214-4 | 40 | 40 | 2 | <0.125 |
| F | 3-2-14 | BC030214-5 | 55 | 55 | 2 | <0.125 |
| M | 3-2-14 | BC030214-6 | 35 | 35 | 2 | <0.125 |
| M | 3-2-14 | BC030214-7 | 30 | 30 | 2 | <0.125 |
| M | 3-2-14 | BC030214-8 | 45 | 45 | 2 | <0.125 |
| F | 3-2-14 | BC030214-9 | 55 | 55 | 2 | <0.125 |
| M | 3-2-14 | BC030214-10 | 40 | 40 | 2 | <0.125 |
| F | 3-2-14 | BC030214-11 | 40 | 40 | 2 | <0.125 |
| M | 3-2-14 | BC030214-12 | 35 | 35 | 2 | <0.25 |
| M | 3-2-14 | BC030214-13 | 50 | 50 | 2 | <0.125 |
| F | 3-2-14 | BC030214-14 | 40 | 40 | 2 | <0.125 |
| M | 3-2-14 | BC030214-15 | 40 | 40 | 2 | <0.25 |
| F | 3-2-14 | BC030214-16 | 50 | 50 | 2 | <0.125 |
| F | 3-2-14 | BC030214-17 | 35 | 35 | 2 | 1.0 |
| M | 3-2-14 | BC030214-18 | 35 | 35 | 2 | <0.25 |
| M | 3-2-14 | BC030214-19 | 30 | 30 | 2 | <0.125 |
| F | 3-2-14 | BC030214-20 | 30 | 30 | 2 | <0.25 |
| M | 3-2-14 | BC030214-21 | 21 | 21 | 2 | <0.25 |
| M | 3-2-14 | BC030214-22 | 30 | 30 | 2 | <0.125 |
| M | 3-2-14 | BC030214-23 | 50 | 50 | 2 | <0.25 |
| F | 3-2-14 | BC030214-24 | 45 | 45 | 2 | <0.25 |
| F | 3-2-14 | BC030214-25 | 60 | 60 | 2 | <0.25 |
| M | 3-2-14 | BC030214-26 | 30 | 30 | 2 | 0.25 |
| M | 3-2-14 | BC030214-27 | 25 | 25 | 2 | <0.25 |
| F | 3-2-14 | BC030214-28 | 25 | 25 | 2 | <0.25 |
| F | 3-2-14 | BC030214-29 | 40 | 40 | 2 | <0.125 |
| M | 3-2-14 | BC030214-30 | 80 | 80 | 2 | <0.25 |
| M | 3-2-14 | BC030214-31 | 35 | 35 | 2 | <0.125 |
| F | 3-2-14 | BC030214-32 | 55 | 55 | 2 | <0.125 |
| F | 3-9-14 | BC030914-1 | 55 | 55 | 2 | <0.125 |
| M | 3-9-14 | BC030914-2 | 50 | 50 | 2 | <0.125 |
| M | 3-9-14 | BC030914-3 | 55 | 55 | 2 | <0.25 |
| F | 3-9-14 | BC030914-4 | 40 | 40 | 2 | <0.25 |
| F | 3-9-14 | BC030914-5 | 30 | 30 | 2 | <0.25 |
| M | 3-9-14 | BC030914-6 | 50 | 50 | 2 | <0.125 |
| F | 3-9-14 | BC030914-7 | 40 | 40 | 2 | 0.25 |
| F | 3-9-14 | BC030914-8 | 50 | 50 | 2 | <0.25 |
| F | 3-9-14 | BC030914-9 | 40 | 40 | 2 | <0.25 |
| M | 3-9-14 | BC030914-10 | 40 | 40 | 2 | <0.125 |
| F | 3-9-14 | BC030914-11 | 50 | 50 | 2 | <0.125 |
| M | 3-9-14 | BC030914-12 | 50 | 50 | 2 | <0.25 |
| F | 3-9-14 | BC030914-13 | 50 | 50 | 2 | <.0125 |
| F | 3-9-14 | BC030914-14 | 60 | 60 | 2 | <0.25 |
| F | 3-9-14 | BC030914-15 | 50 | 50 | 2 | <0.25 |
